# Severe and Prolonged Paroxetine Intoxication With Delayed Absorption and Extreme Serum Concentrations: A Case Report

**DOI:** 10.7759/cureus.117066

**Published:** 2026-09-28

**Authors:** Luna Beijnsberger, Chantal A Boly, Tessa C Jaspers, Jos A Van Oers

**Affiliations:** 1 Intensive Care Medicine, Elisabeth-TweeSteden Ziekenhuis, Tilburg, NLD; 2 Clinical Pharmacy, Elisabeth-TweeSteden Ziekenhuis, Tilburg, NLD

**Keywords:** acute respiratory distress syndrome (ards), clinical pharmacokinetics, drug-induced toxicity, paroxetine, serotonin syndrome (ss)

## Abstract

Selective serotonin reuptake inhibitors (SSRIs) are commonly prescribed antidepressants, known for their relatively safe pharmacological profile and mild symptoms in cases of overdose compared with other antidepressants. In this case report, we describe a near-fatal case of a 70-year-old woman who ingested multiple drugs with suicidal intent, including an unknown quantity of celecoxib 200 mg, clorazepate acid 5 mg, omeprazole 20 mg, and immediate-release paroxetine 30 mg tablets. The patient developed severe serotonin syndrome complicated by acute respiratory distress syndrome (ARDS) and required admission to the intensive care unit (ICU). Because of progressive respiratory deterioration, she required intubation and invasive mechanical ventilation. Serotonin syndrome persisted for an unusually long period, with serum paroxetine concentrations of 4805 µg/L at admission that continued to rise to a maximum of 7824 µg/L on day four, both well above the lethal threshold. Intensive therapeutic interventions aimed at lowering paroxetine concentrations and resolving serotonin syndrome included activated charcoal, whole-bowel irrigation lavage, and rifampicin. Paroxetine concentrations gradually declined and reached the therapeutic range after 16 days. Following further ICU treatment, including prone ventilation and corticosteroids, the ARDS resolved, and the patient was extubated 21 days after ingestion. This case highlights that paroxetine overdose may present more severely than commonly reported in the literature, with delayed and prolonged toxicity, including serotonin syndrome and ARDS. Paroxetine overdose may be life-threatening and underscores the need for close monitoring of serum paroxetine concentrations and timely consideration of activated charcoal and whole-bowel irrigation.

## Introduction

Paroxetine is a selective serotonin reuptake inhibitor (SSRI). SSRIs are the most frequently used antidepressants. The mechanism of action involves inhibition of the presynaptic uptake of serotonin in the central nervous system, resulting in prolonged stimulation of postsynaptic serotonin receptors [1]. SSRI overdoses are generally considered relatively safe and rarely fatal, typically presenting with mild to moderate symptoms such as nausea, vomiting, dizziness, and mild central nervous system depression compared to other antidepressants [2,3]. Serious complications are rare and usually associated with concomitant medications that enhance serotonin concentrations, such as a monoamine oxidase inhibitor, or after ingestion of extreme doses [2]. Paroxetine is rapidly absorbed with a time to maximum serum concentration (Tmax) of approximately six hours and undergoes extensive hepatic metabolism, primarily via cytochrome P450 (CYP) pathways, especially CYP2D6. In therapeutic use, paroxetine has a relatively short elimination half-life of approximately one day [4,5].

We present a case of a severe, near-fatal, immediate-release paroxetine overdose, complicated by the development of serotonin syndrome, a potentially life-threatening condition caused by excessive serotonergic activity, and acute respiratory distress syndrome (ARDS), a severe inflammatory lung injury causing non-cardiogenic respiratory failure, and describe the pharmacokinetics of paroxetine and the management of paroxetine intoxication during the patient’s intensive care unit (ICU) stay. This case highlights the potential lethal complications of an SSRI overdose and demonstrates that clinical deterioration may occur over several days rather than hours.

This article was previously presented as a meeting abstract at the Dutch Society of Critical Care Medicine Annual Scientific Meeting on February 5, 2026.

## Case presentation

A 70-year-old woman with a body weight of 55 kg and a medical history of depression, alcohol use disorder, and hypothyroidism presented to the emergency department in June 2025 with an altered level of consciousness. The patient was found in bed, with multiple empty medication containers at her bedside. The medications identified included celecoxib 200 mg, clorazepate acid 5 mg, paroxetine 30 mg, and omeprazole 20 mg. The number of tablets ingested of each medication ingested during the suicidal attempt was unknown. Upon admission, approximately 12 hours after the suspected time of ingestion, her vital signs were as follows: Glasgow Coma Scale (GCS) E4M6V2 [6], without midriasis, blood pressure 144/83 mmHg, heart rate 84 beats per minute, respiratory rate 17 breaths per minute, peripheral oxygen saturation 93% without supplemental oxygen, and a body temperature of 36.8 °C. The patient exhibited coarse breath sounds, with rhonchi in the right lower lung fields, raising suspicion for aspiration pneumonia. Further neurological examination revealed reduced attention span, hypophonic speech, myoclonus affecting the hands, legs, and head, tremor of the hands, and hyperreflexia. Blood tests revealed normal renal function, electrolyte levels, and liver enzymes (Table 1). The white blood cell count was mildly elevated at 12.2 x 10⁹/L. A chest radiograph revealed pulmonary congestion and infiltrative abnormalities in both lower lung fields (Figure 1). An electrocardiogram (ECG) showed a tachycardic sinus rhythm with a prolonged QTc interval of 647ms, without signs of ischemia (Figure 2). As the expected Tmax of paroxetine, celecoxib, clorazepate acid, and omeprazole had presumably already elapsed, no activated charcoal was given. The clinical manifestations and prolonged QTc interval were most consistent with effects of paroxetine. It was unlikely that co‑ingestants or electrolyte abnormalities explained the prolonged QTc. The patient was admitted to the ICU because of the risk of cardiac side effects, serotonin syndrome, apneas, and convulsions.

**Table 1:** Laboratory examination at presentation AST - aspartate transferase; ALT - alanine transaminase; γ-GTP - γ-glutamyl transpeptidase; ALP - alkaline phosphatase

Parameter	Patient value	Reference range
Hemoglobin	13.5 g/dL	13.7-17.7 g/dL
Hematocrit	0.41 L/L	0.4-0.5 L/L
White blood cell count	12.2 x 10^9^/L	4.0-10.0 x 10^9^/L
Platelet	220 x 10^9^/L	150-400 x 10^9^/L
Blood urea nitrogen	20.4 mg/dL	8.1-21.0 mg/dL
Creatinine	0.71 mg/dL	0.72-1.18 mg/dL
Sodium	142 mmol/L	135-145 mmol/L
Potassium	4.3 mmol/L	3.5-5.0 mmol/L
Magnesium	0.96 mmol/L	0,7-1,1 mmol/L
Total calcium	2.31 mmol/L	2,20-2.65 mmol/L
Albumin	47 g/L	35-50 g/L
AST	30 U/L	< 31 U/L
ALT	33 U/L	< 35 U/L
γ-GTP	12 U/L	< 40 U/L
ALP	58 U/L	33-98 U/L

**Figure 1:**
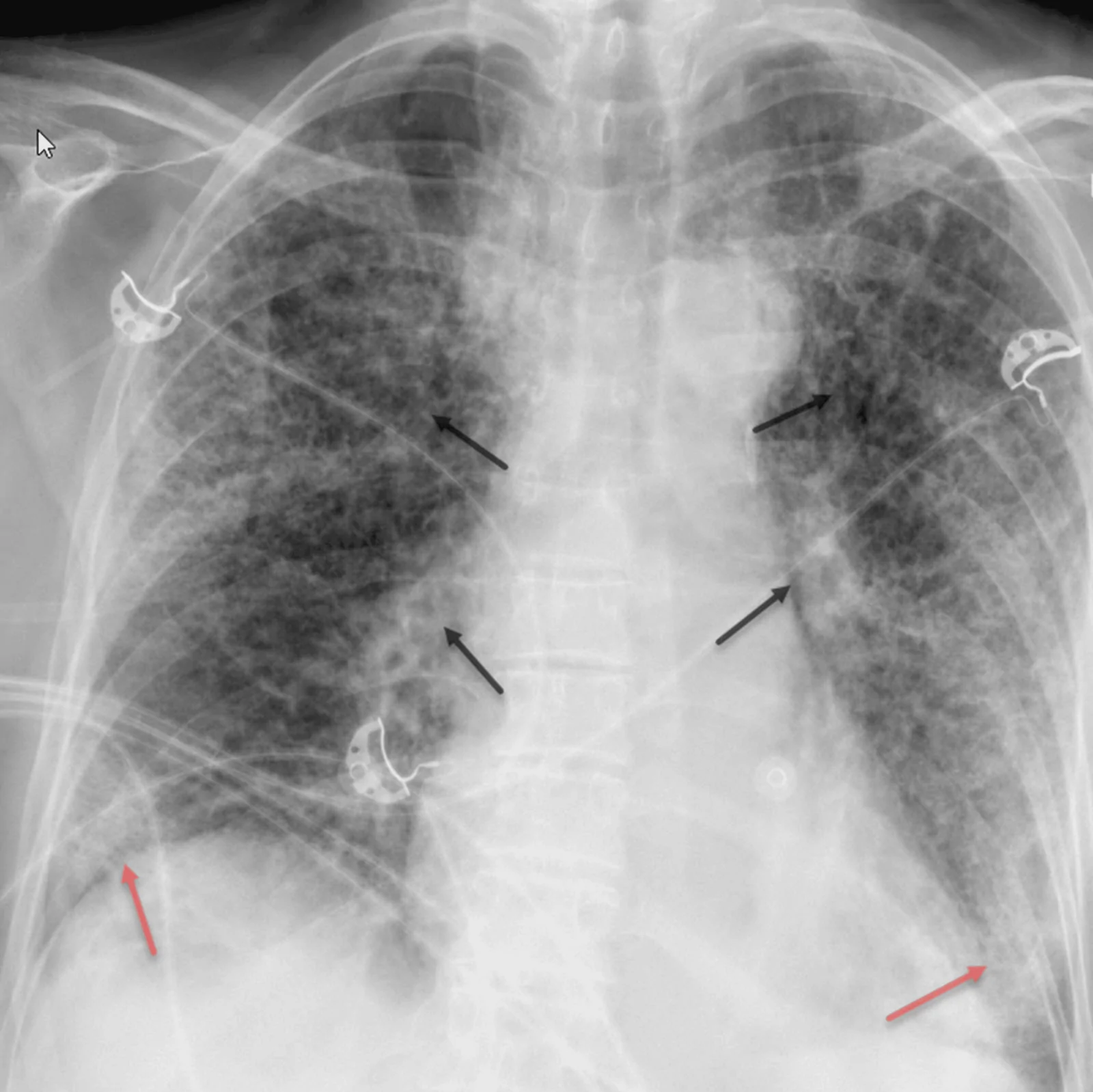
Chest radiograph at presentation Red arrows indicate consolidative infiltrates in the lower lung fields, black arrows indicate pulmonary congestion.

**Figure 2:**
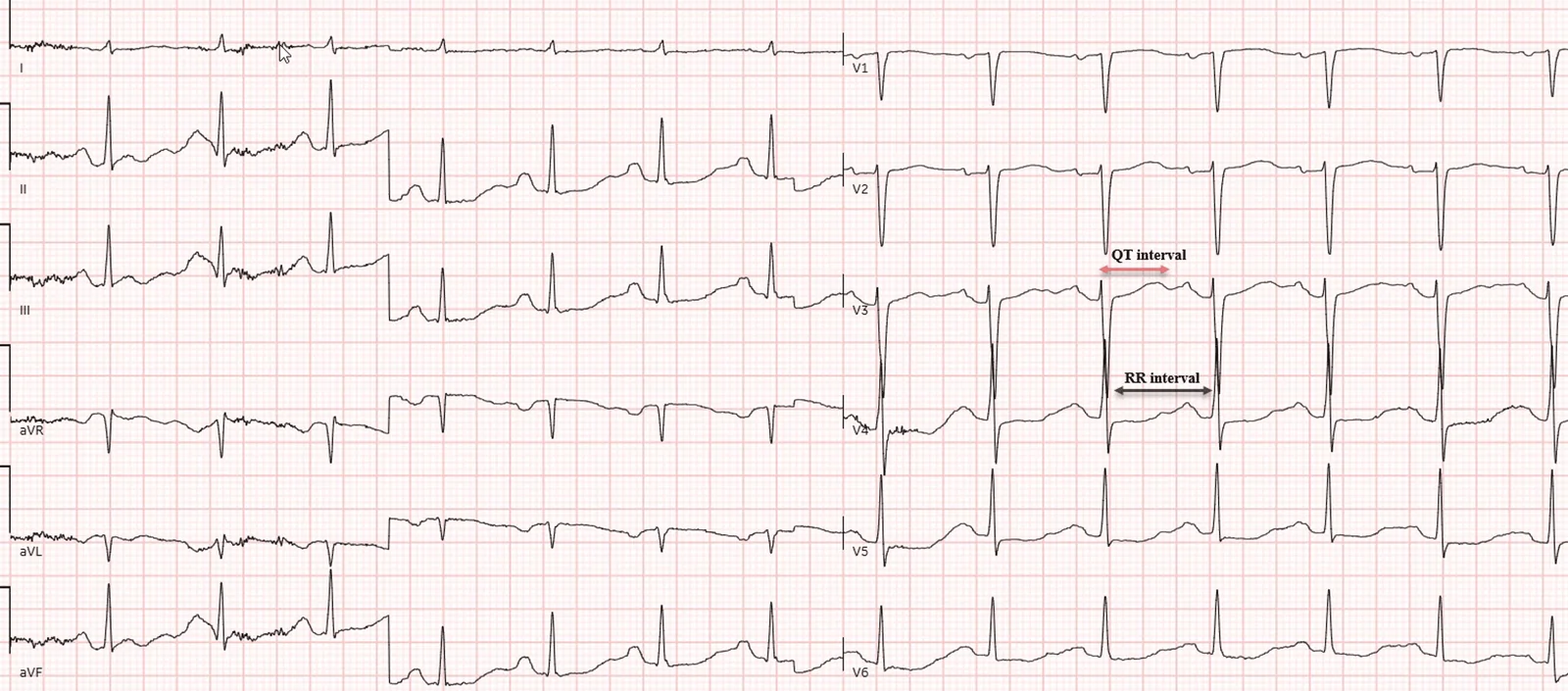
Electrocardiogram at presentation Red arrow = QT interval; black arrow = RR interval; QTc (s) = QT÷ √RR;  QT interval = 0.528 s; RR interval = 0.666 s; QTc = 0.647 s

On day two of admission, the neurological status remained unchanged. The temperature was still normal. However, the respiratory state of the patient worsened. A new chest radiograph showed enhanced vascular markings. Lung ultrasound showed strong B-lines. Despite administration of furosemide, the respiratory state did not improve. Due to persistent reduced attention span, somnolence, myoclonus, prolonged QTc, and no defecation since admittance to the ICU, ongoing paroxetine absorption due to the anticholinergic effects of a massive paroxetine overdose was suspected. Therefore, on day three, repeated doses (50 gram, every four hours) of activated charcoal were given until the stool turned black. Non-invasive ventilation (NIV) was initiated on day four of admission due to hypoxemia. The patient subsequently required intubation because of worsening respiratory failure and intolerance of NIV. After intubation, she developed hyperthermia with a temperature of 40.6°C without any signs suggestive of an infection. The fever was not attributed to intubation, sedation, or neuromuscular blockade. As systolic blood pressure fell below 90 mm Hg, a minimal dose of norepinephrine up to 0.23 mcg/kg/min was required to maintain blood pressure. However, there was no increase in heart rate, the patient's skin was well-perfused without evidence of mottling, and urine output remained within normal limits. Neurological examination was not possible due to deep sedation. A computerized tomography scan (CT) of the thorax and abdomen was performed, which showed no infiltrates or pharmacobezoars. Given the combination of a massive paroxetine overdose, myoclonus, tremor, hyperreflexia, and subsequent hyperthermia, serotonin syndrome was diagnosed according to the Sternbach criteria [7]. However, empirical treatment with ceftriaxone was initiated as a precaution for a potential bacterial pneumonia.

The serum paroxetine concentrations over time are shown in Figure 3. Concentrations were initially measured retrospectively on day four. On the day of admission, the serum paroxetine concentration was 4,805 µg/L (therapeutic range 20-200 µg/L) and continued to rise to 7824 µg/L on day four, well above the reported lethal concentration of 4000 µg/L [5] and well beyond the reported Tmax of six hours [5]. Despite the repeated administration of activated charcoal on day three, drug concentrations increased further on day four. Therefore, whole-bowel irrigation was initiated on day five of admission due to suspected pharmacobezoars, despite the absence of visible pharmacobezoars on abdominal CT. From day five onward, circulating paroxetine concentrations declined, with an average elimination half-life of 46.1 hours between day four and 18. However, measured concentrations slightly increased between day six and eight. We did not measure concentrations of celecoxib, omeprazole, or clorazepate acid because these intoxications were considered clinically mild and the presentation was consistent with paroxetine intoxication.

**Figure 3:**
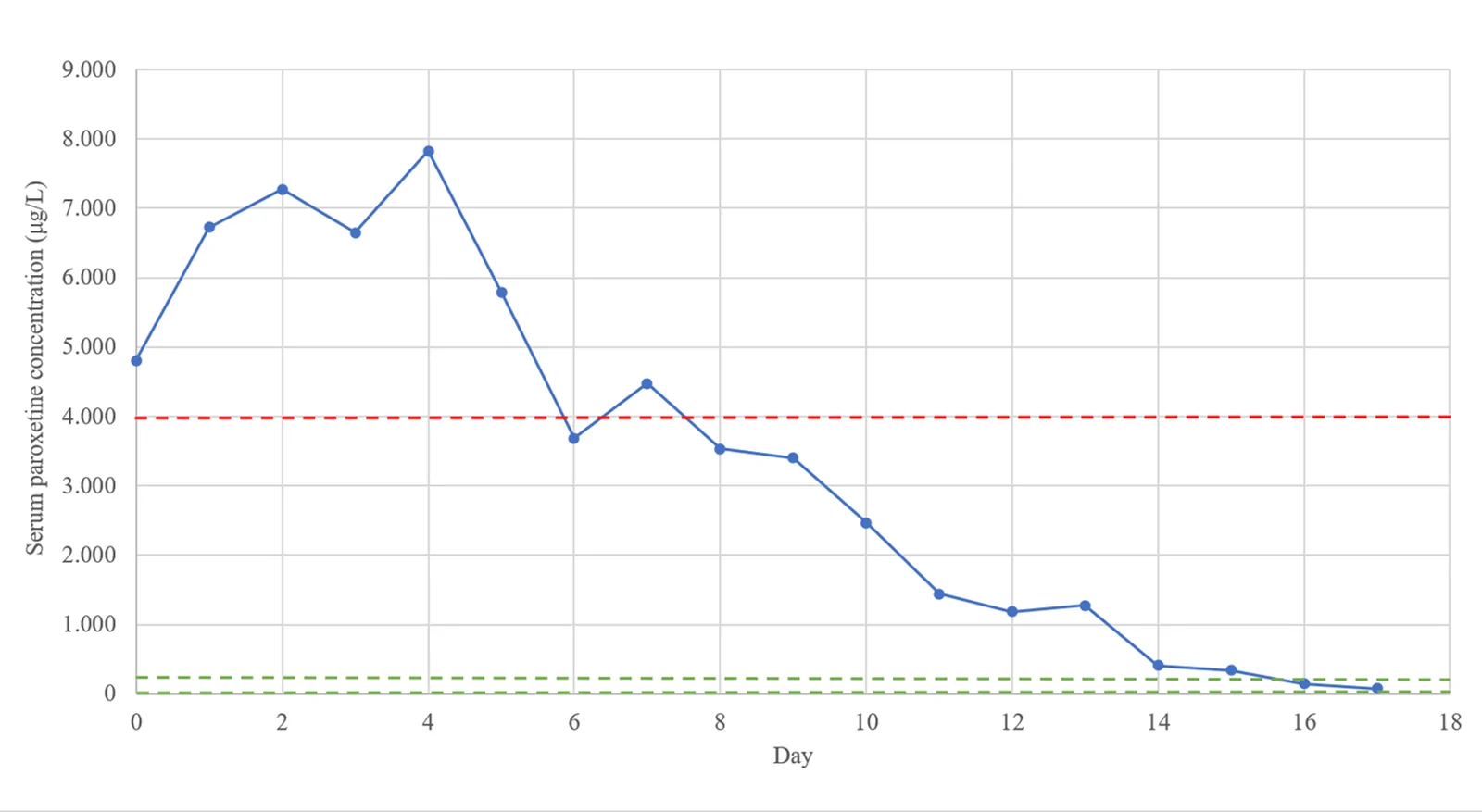
Serum paroxetine concentrations over time Red dashed line represents the lethal concentration; green dashed lines represent the therapeutic range. Day three started activated charcoal; day five started whole bowel irrigation; day six started rifampicin.

Because of a low PaO2/FiO2 (P/F) ratio of 103, the patient was turned in prone position on day six. On day six, treatment with rifampicin 600 mg twice daily was initiated to increase the metabolism of paroxetine, given the reported half-lives of approximately 200 hours in paroxetine intoxications [8]. Ventilatory settings and the level of hemodynamic support could be reduced. On day ten, the patient's respiratory condition worsened again. Increased sedation was required, and the ventilatory settings were adjusted from pressure support ventilation to pressure-controlled ventilation. The patient was turned in prone position again, but showed no significant response. A CT scan of the thorax showed increased bilateral consolidations, persistent enhancement of the interstitial pattern, and bilateral subsegmental pulmonary emboli, without signs of elevated right-sided pressures (Figure 4). Therapeutic anticoagulation with nadroparin was initiated. A bronchoalveolar lavage (BAL) was performed, which showed no evidence of bacterial growth or viral presence. Acute respiratory distress syndrome (ARDS) was suspected. Methylprednisolone infusion was initiated at a dose of 2 mg/kg/day. 

**Figure 4:**
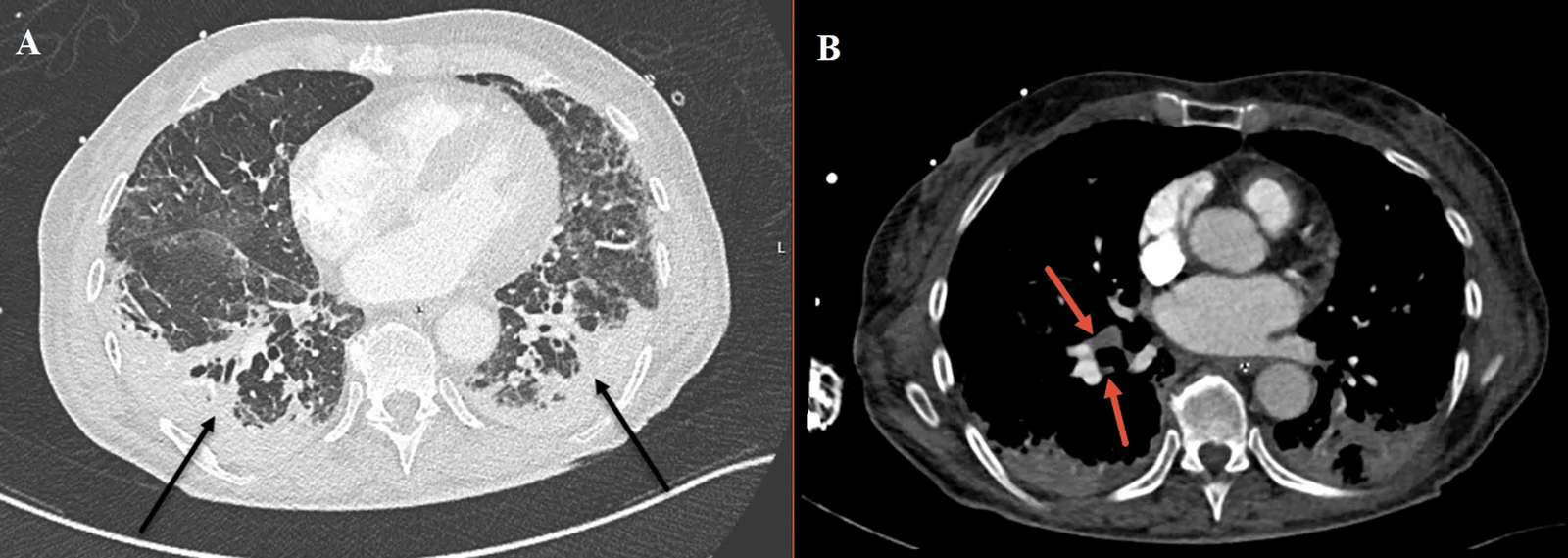
CT scan of the thorax A) Bibasal consolidations (black arrows); B) Subsegmental pulmonary embolism in the right lung (red arrows)

On day 15, the patient became more alert, and the ventilation settings were adapted to pressure support as oxygen and compliance improved. On day 21, the patient was extubated, and high-flow oxygen was started. Because of normal paroxetine concentrations, rifampicin could be stopped on day 22. The patient was discharged from the ICU after 25 days. During her subsequent stay on the medical ward, she developed hyperactive delirium, which was considered unrelated to the serotonin syndrome and was treated with haloperidol. At hospital discharge on day 46, the patient was hemodynamically and respiratory stable, with complete resolution of the serotonin syndrome and ARDS. However, she had persistent profound muscle weakness and fatigue and therefore required transfer to a nursing home for further rehabilitation. No further serum paroxetine measurements were available. Long-term follow-up after transfer to the nursing home was unavailable.

## Discussion

This case highlights a rare and severe presentation of an immediate-release paroxetine overdose, distinguished by the following features: development of serotonin syndrome, ARDS, and prolonged toxic paroxetine concentrations beyond the expected time to maximum serum concentration and elimination half-life. The temporal relationship (possible Tmax up to 96 hours) and biological plausibility (spontaneous myoclonus, hyperreflexia) supported a possible causal association between clinical findings and paroxetine overdose in the causality assessment. SSRI intoxications are generally considered to have a relatively benign course. However, paroxetine may present with more pronounced toxicity, as seen in our patient. To our knowledge, no case reports have described paroxetine serum concentrations as high as those observed in our case. Notably, the patient survived despite these extreme concentrations, whereas fatal outcomes have been reported at lower concentrations [9].

Serotonin syndrome was diagnosed according to the Sternbach criteria, based on recent exposure to a serotonergic agent and the presence of myoclonus, tremor, hyperreflexia, and subsequent hyperthermia [7]. The incidence of serotonin syndrome varies, but it is generally considered rare in cases of isolated paroxetine overdose. Treatment typically involves discontinuing the offending medication, providing supportive care, and the administration of benzodiazepines [9]. 

The etiology of ARDS in this case remains uncertain. Although aspiration pneumonia was considered a possible cause, microbiological investigations did not support this diagnosis, as cultures of sputum, bronchoalveolar lavage fluid, and blood showed no bacterial growth. Therefore, paroxetine toxicity may also have contributed to the development of ARDS. A few case reports have described an association between ARDS and SSRI use [9,10-12]. One case report described fatal toxicity following a massive overdose with long-acting paroxetine [9], while another reported pulmonary edema at postmortem examination after paroxetine overdose [10]. In addition, diffuse alveolar damage (DAD) has been described as an adverse effect of paroxetine and sertraline even in the absence of overdose or intoxication [11,12]. Pulmonary emboli may also have contributed to the respiratory failure, although their origin remains uncertain.

The serum paroxetine concentrations of this patient were extremely high and continued to rise beyond the expected kinetics. At admission, the measured concentration in our patient was 4805 µg/L, exceeding reported lethal thresholds of 4000 µg/L in overdoses [5]. This concentration continued to rise, reaching 7824 µg/L by day four, before beginning to decrease. This pattern does not fit the expected kinetics and is suggestive of ongoing absorption. To our knowledge, such delayed absorption has not previously been described in the absence of pharmacobezoar formation. This observation is clinically relevant as it suggests that, in overdoses with potent anticholinergic agents, or other drugs that impair gastrointestinal motility such as opioids, a markedly delayed Tmax should be considered.

In this case, repeated doses of activated charcoal were given until the stool turned black. The 2025 guidance for the administration of activated charcoal states that activated charcoal may be administred beyond the conventional six-hour post-ingestion window when ongoing absorption is suspected, or the ingested drug burden exceeds its solubility limits [13]. After the last dose, the paroxetine concentrations continued to rise. Because of the persistent concern for a pharmacobezoar despite not being visible on CT abdomen, whole-bowel irrigation was initiated on day five. In retrospect, whole-bowel irrigation should have been initiated upon admission, as it can effectively reduce ongoing gastrointestinal absorption [14]. However, whole-bowel irrigation is usually used in cases involving substances that do not bind to activated charcoal, poorly water-soluble substances, or when there is a high risk of pharmacobezoar formation, such as with large ingestions of slow-release medication [15]. Paroxetine does not meet these criteria, and to our knowledge, whole-bowel irrigation has not been reported in case reports describing a paroxetine intoxication. However, the abrupt decline in paroxetine serum concentrations after day five was likely due to reduced ongoing gastrointestinal absorption after repeated activated charcoal administration and whole-bowel irrigation, suggesting a persistent intraluminal drug reservoir.

In addition to delayed absorption, other mechanisms may have contributed to the prolonged elevated serum concentrations and markedly prolonged half-life, including saturable first-pass metabolism, redistribution due to the large volume of distribution of paroxetine, autoinhibition of CYP2D6 metabolism, and altered distribution during critical illness. Normally, paroxetine has a bioavailability of 30-60% due to extensive first-pass metabolism. In a paroxetine overdose, these pathways may become saturated, increasing systemic exposure [16]. Moreover, its lipophilic nature and large volume of distribution may lead to redistribution from peripheral compartments, contributing to prolonged serum concentrations [16]. This might also explain the rise in serum concentration after clearance by the intestines due to repeated doses of activated charcoal on day four and may explain the rise in serum concentrations between days six and eight. Assay variability may also have contributed to the observed rise in serum concentration. Paroxetine is metabolized in the liver by CYP2D6 enzymes. In addition, it also inhibits these enzymes, leading to reduced clearance [17]. This inhibition of CYP2D6 may have been enhanced by concomitant celecoxib exposure, which can also inhibit CYP2D6 [18]. Critical illness, through alterations in plasma protein binding and tissue perfusion, as well as therapeutic interventions such as fluid resuscitation, can significantly affect the volume of distribution, serum drug concentration, and drug delivery to the tissues [19].

Because the patient's clinical condition deteriorated significantly, we chose to initiate rifampicin to accelerate metabolism on day six. Although rifampicin induces CYP enzymes, including CYP2D6, its effect typically occurs after several days [20]. This is not standard treatment for paroxetine intoxication. In this case, no clear effect on paroxetine metabolism was observed, possibly because serum concentrations had already begun to decline from day four.

A strength of this case is the detailed clinical description combined with extensive pharmacokinetic data and evaluation. Serial paroxetine measurements allowed a clear characterization of the serum concentration-time course of paroxetine, providing insights into the complex pharmacokinetics in overdose. However, several limitations should be acknowledged. Paroxetine concentrations were measured externally, resulting in retrospective availability of concentrations on day four. Subsequent measurements were performed every three to five days, which limited real-time clinical decision-making. As a result, certain treatment decisions might have differed if daily measurements were available. For example, rifampicin might not have been initiated if the declining trend in paroxetine concentrations had been recognized earlier. This case underscores that, even in drugs generally considered safe, pharmacokinetic unpredictability in overdose can lead to delayed and severe toxicity, necessitating prolonged ICU admission. 

## Conclusions

This case report describes severe paroxetine intoxication complicated by serotonin syndrome and ARDS. Pharmacokinetic findings revealed persistently extreme serum paroxetine concentrations, likely attributable to delayed absorption and multiple interacting pharmacokinetic mechanisms. The unusually prolonged toxicity and extreme serum concentrations observed in this case highlight the potential for paroxetine overdose to result in a protracted clinical course and underscore the value of extended pharmacokinetic monitoring and early gastrointestinal decontamination measures, including activated charcoal and whole bowel irrigation.

## References

[1] Kowalska M, Nowaczyk J, Fijałkowski Ł, Nowaczyk A (2021). Paroxetine-overview of the molecular mechanisms of action. Int J Mol Sci.

[2] Nelson LS, Erdman AR, Booze LL (2007). Selective serotonin reuptake inhibitor poisoning: an evidence-based consensus guideline for out-of-hospital management. Clin Toxicol (Phila).

[3] Barbey JT, Roose SP (1998). SSRI safety in overdose. J Clin Psychiatry.

[4] Boyer WF, Feighner JP (1992). An overview of paroxetine. J Clin Psychiatry.

[5] Schulz M, Schmoldt A, Andresen-Streichert H, Iwersen-Bergmann S (2020). Revisited: therapeutic and toxic blood concentrations of more than 1100 drugs and other xenobiotics. Crit Care.

[6] Teasdale G, Jennett B (1974). Assessment of coma and impaired consciousness: a practical scale. Lancet.

[7] Sternbach H (1991). The serotonin syndrome. Am J Psychiatry.

[8] Hilleret H, Voirol P, Bovier P (2002). Very long half-life of paroxetine following intoxication in an extensive cytochrome P4502D6 metabolizer. Ther Drug Monit.

[9] Inoue F, Okazaki Y, Ichiba T, Kashiwa K, Namera A (2023). Unexpectedly prolonged serotonin syndrome and fatal complications following a massive overdose of paroxetine controlled-release. Cureus.

[10] Motonori T, Hiroshi K, Azumi K (2010). An autopsy case of poisoning with selective serotonin reuptake inhibitor, paroxetine. Soud Lek.

[11] Antonello N, Sergio L, Andrea T, Cornelius B (2015). Pulmonary drug toxicity: presentation of a case of recurrent diffuse alveolar damage caused by paroxetine. Am J Ther.

[12] Torok NI, Donaldson BL, Taji J, Abugiazya A, Assaly R (2012). Diffuse alveolar damage and recurrent respiratory failure secondary to sertraline. Am J Ther.

[13] Hoegberg LC, Gosselin S, Buckley NA (2026). Recommendations from the clinical toxicology recommendations collaborative on the administration of activated charcoal in acute oral overdose. Clin Toxicol (Phila).

[14] Tenenbein M, Cohen S, Sitar DS (1987). Whole bowel irrigation as a decontamination procedure after acute drug overdose. Arch Intern Med.

[15] Sandilands EA (2023). Whole-bowel irrigation should not be used routinely in poisoned patients. Br J Clin Pharmacol.

[16] Kaye CM, Haddock RE, Langley PF, Mellows G, Tasker TC, Zussman BD, Greb WH (1989). A review of the metabolism and pharmacokinetics of paroxetine in man. Acta Psychiatr Scand Suppl.

[17] Damborská A, Hanáková L, Pindurová E, Horská K (2024). Case report: therapeutic drug monitoring and CYP2D6 phenoconversion in a protracted paroxetine intoxication. Front Pharmacol.

[18] Werner U, Werner D, Rau Th, Fromm MF, Hinz B, Brune K (2003). Celecoxib inhibits metabolism of cytochrome P450 2D6 substrate metoprolol in humans. Clin Pharmacol Ther.

[19] Smith BS, Yogaratnam D, Levasseur-Franklin KE, Forni A, Fong J (2012). Introduction to drug pharmacokinetics in the critically ill patient. Chest.

[20] Polasek TM, Lin FP, Miners JO, Doogue MP (2011). Perpetrators of pharmacokinetic drug-drug interactions arising from altered cytochrome P450 activity: a criteria-based assessment. Br J Clin Pharmacol.

